# Boosting the hypoxic response in myeloid cells accelerates resolution of fibrosis and regeneration of the liver in mice

**DOI:** 10.18632/oncotarget.14749

**Published:** 2017-01-19

**Authors:** Chahrazade Kantari-Mimoun, Ewelina Krzywinska, Magali Castells, Corinne Milien, Ralph Klose, Anna-Katharina Meinecke, Ursula Lemberger, Thomas Mathivet, Milos Gojkovic, Katrin Schrödter, Christoph Österreicher, Joachim Fandrey, Helene Rundqvist, Christian Stockmann

**Affiliations:** ^1^ Institut National de la Santé et de la Recherche Médicale (INSERM), Unit 970, Paris Cardiovascular Research Center, Paris, France; ^2^ Institut für Physiologie, Universitätsklinikum Essen, Universität Duisburg-Essen, Germany; ^3^ Division of Gastroenterology and Hepatology Department of Medicine III Medical University of Vienna, Austria; ^4^ Department of Cell and Molecular Biology, Karolinska Institutet, Stockholm, Sweden; ^5^ Institute of Pharmacology, Center for Physiology and Pharmacology Medical University of Vienna, Austria

**Keywords:** liver fibrosis, vascular endothelial growth factor, scar-associated macrophage, matrix metalloproteinases, fibrolysis

## Abstract

We have recently shown that targeting Vascular Endothelial Growth Factor (VEGF) specifically in scar-infiltrating myeloid cells prevented remodeling of the sinusoidal vasculature and abrogated the resolution of murine liver fibrosis, thereby unmasking an unanticipated link between angiogenesis and resolution of fibrosis. In a gain of function approach, we wanted to test the impact of VEGF overexpression in myeloid cells on fibrolysis. We observe that genetic inactivation of the von Hippel Lindau protein (VHL), a negative regulator of Hypoxia-inducible factors (HIF) in myeloid cells, leads to increased VEGF expression and most importantly, accelerated matrix degradation and reduced myofibroblast numbers after CCl_4_ challenge. This is associated with enhanced expression of MMP-2 and -14 as well as lower expression of TIMP-2 in liver endothelial cells. In addition, we report increased expression of MMP-13 in scar-associated macrophages as well as improved liver regeneration upon ablation of VHL in myeloid cells. Finally, therapeutic infusion of macrophages nulli-zygous for VHL or treated with the pharmacologic hydroxylase inhibitor and HIF-inducer Dimethyloxalylglycine (DMOG) accelerates resolution of fibrosis. Hence, boosting the HIF-VEGF signaling axis in macrophages represents a promising therapeutic avenue for the treatment of liver fibrosis.

## INTRODUCTION

Liver fibrosis is characterized by the loss of parenchyma and deposition of extracellular matrix (ECM) components by activated myofibroblasts, ultimately leading to excessive scar formation and organ failure [1, 2]. However, liver fibrosis in response to chronic liver injury is now considered as a dynamic and reversible process, even at advanced stages [3]. The recovery process requires first, ECM degradation along with resolution of the fibrotic scar, and second, regeneration of the hepatocyte population involving the activation of liver progenitor cells [4, 5]. The monocyte-macrophage lineage plays a crucial role in the resolution of fibrosis as well as liver regeneration by driving the expansion of liver progenitor cells [6]. In this context, different macrophage subtypes have been described including antifibrotic, CD11B^hi^ F4/80^int^ Ly6C^lo^ “restorative” macrophages with crucial pro-resolution properties [7]. Therefore, bone marrow cell treatment for liver cirrhosis holds considerable therapeutic potential [4, 6, 8].

We have recently reported that targeting vascular endothelial growth factor (VEGF) specifically in scar-infiltrating myeloid cells prevented remodeling of the sinusoidal vasculature and abrogated the resolution of murine liver fibrosis. Furthermore, we showed that the resolution of liver fibrosis is associated with increased expression of matrix metalloproteases (MMP)-2 and -14 as well as decreased expression of tissue inhibitor of metalloproteases (TIMP)-1 and 2 confined to sinusoidal endothelium, thereby unmasking an unanticipated link between angiogenesis and resolution of fibrosis [9].

VEGF expression is at least partially regulated by low oxygen levels through a transcription factor family called Hypoxia-inducible factors (HIF) [10]. HIFs are basic-helix-loop-helix transcription factors that consist of a constitutively expressed β-subunit and a regulatory α-subunit. α-subunits are hydroxylated by prolyl hydroxylases in the presence of oxygen and subsequently degraded through the ubiquitin proteasome pathway via interaction with their negative regulator von Hippel-Lindau (VHL) protein [11, 12]. Here, we tested the therapeutic potential of a gain of function approach by genetically inactivating the von Hippel Lindau protein (VHL), a negative regulator of HIF and HIF-dependent VEGF expression [10, 11] specifically in myeloid cells (VHL^fl/fl^-LysMCre+ mice), which results in VEGF overexpression in scar-associated macrophages. We observed that transplantation of bone marrow (BM) from VHL^fl/fl^-LysMCre+ mice into C57Bl6/J mice accelerates fibrosis resolution and reduces the myofibroblast population after carbontetrachloride- (CCl_4_) challenge. This was associated with enhanced expression of MMP-2 and -14 in sorted liver endothelial cells, thus further substantiating the role of VEGF as a driver of fibrolysis [9, 13]. We report increased expression of MMP-7 and -9 in whole livers along with an upregulation of MMP-13 in liver macrophages after reconstitution with VHL^fl/fl^-LysMCre+ bone marrow. Furthermore, reconstitution with VHLfl/fl-LysMCre+ bone marrow results in increased number of pancytokeratin- and Dlk-expressing liver progenitor cells, indicating improved liver regeneration along with accelerated fibrolysis. Noteworthy, simultaneous deletion of VEGF and VHL in myeloid cells (VHL^fl/fl^/VEGF^fl/fl^-LysMCre+ mice) largely reversed the phenotype observed in VHL^fl/fl^-LysMCre+ mice. Finally, macrophage therapy by infusion of either VHL-deficient macrophages or macrophages treated with the pharmacologic prolyl-hydroxylase inhibitor and HIF-inducer Dimethyloxalylglycine (DMOG) in fibrotic mice enhanced scar resolution. Given the current efforts of macrophage-based therapy for liver fibrosis, we propose that boosting the hypoxic response in scar infiltrating macrophages could represent a therapeutic avenue for the treatment of liver fibrosis and improvement of liver regeneration.

## RESULTS

### Genetic targeting of VHL in myeloid cells increases VEGF expression and accelerates the resolution of fibrosis

**Figure 1:**
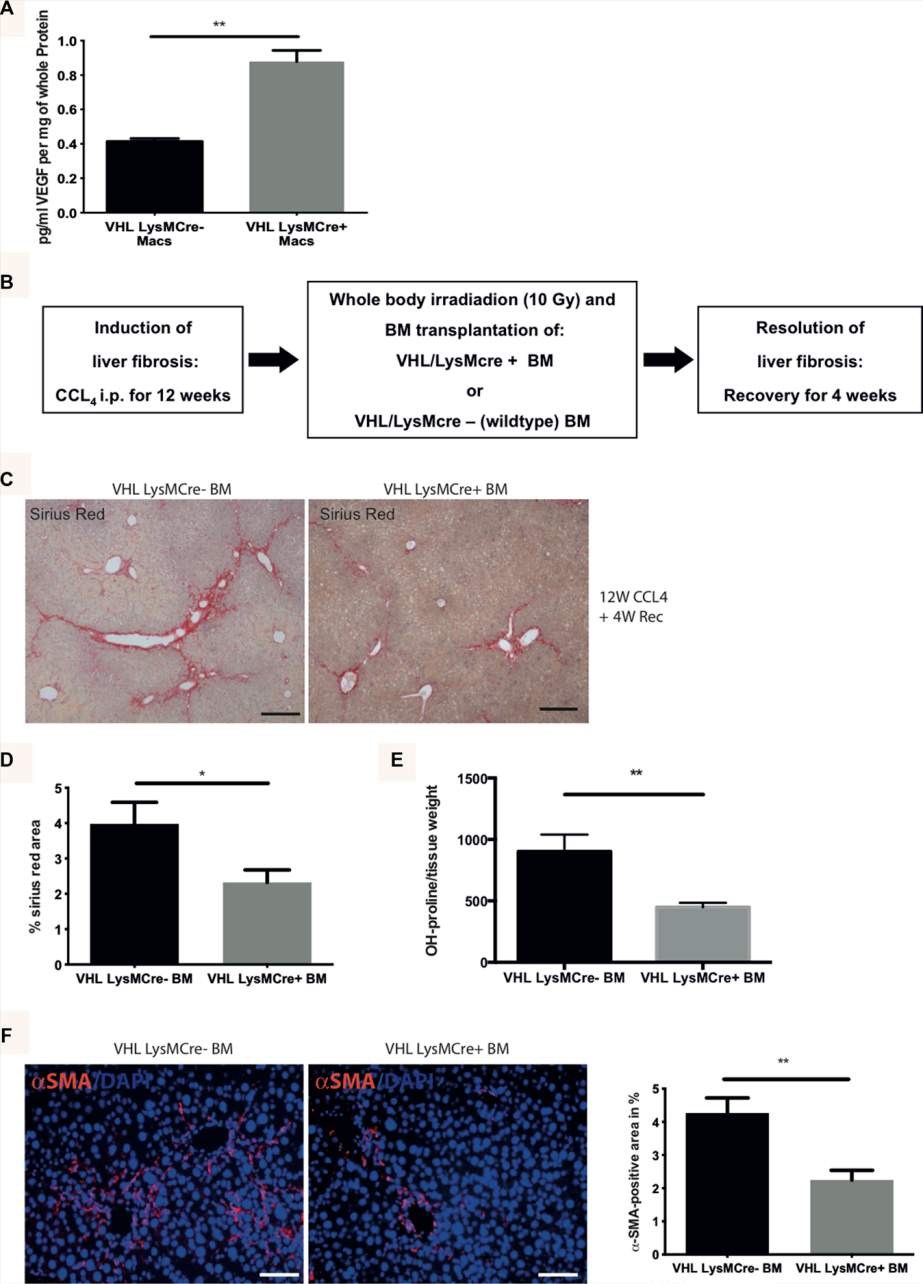
Transplantation of bone marrow from VHL^fl/fl^-LysMCre+ mice into C57Bl6/J mice after CC_l4-_challenge accelerates fibrosis resolution as compared to mice after reconstitution with wildtype (VHL^fl/fl^-LysMCre-) bone marrow (**A**) VEGF release by macrophages (Macs) from VHL^fl/fl^-LysMCre- (*n* = 5) and VHL ^fl/fl^- LysMCre+ mice (*n* = 6). (**B**) Time-schedule for CCl4-treatment, bone-marrow transplantation and subsequent fibrosis resolution. (**C**) Representative histological images of Sirius Red-stained liver sections from mice after reconstitution with bone marrow (BM) from VHL LysMCre- (left panel) and VHL LysMCre+ mice (right panel) after 12 weeks of treatment with CCl4 and 4 weeks of recovery. (**D**) Quantification of Sirius Red-positive area on murine livers sections (*n* = 5 for VHL LysMCre- and *n* = 7 for VHL LysMCre+). (**E**) Determination of free hydroxyproline in liver tissue samples (*n* = 5 for VHL LysMCre- and *n* = 7 for VHL LysMCre+). (**F**) Representative histological images of α-SMA-stained liver sections from the two groups of mice and quantification of α-SMA-positive area (*n* = 5 for VHL LysMCre- and *n* = 7 for VHL LysMCre+). Error bars represent SEM. Scale bars equal 100 μm.

VEGF is emerging as a driver of fibrolysis and we have recently identified myeloid cells as an indispensable source of VEGF upon fibrosis resolution [9, 13]. Here, we tested the therapeutic potential of genetically inactivating the von Hippel Lindau protein (VHL), a negative regulator of Hypoxia-inducible factors (HIF) and HIF-dependent VEGF expression [10, 11] specifically in myeloid cells (VHL^fl/fl^-LysMCre+ mice) using a *loxP*-flanked VHL allele crossed to the lysozyme M promoter-driven Cre recombinase [14] which results in VEGF overexpression in macrophages (Figure 1A). To investigate the impact of this gain of function approach specifically on the resolution of fibrosis, we subjected CCl_4_-treated C57Bl6/J mice to a previously established model [9] of whole body irradiation (10 Gy) and transplantation of bone marrow (BM) from VHL^fl/fl^-LysMCre+ mice or wildtype (VHL^fl/fl^-LysMCre-) mice (as depicted in Figure 1B), followed by a 4 week recovery phase. Our previous studies [9] showed that spontaneous and complete resolution without irradiation is achieved after 4 weeks of recovery whereas the irradiation procedure along with BM reconstitution delays the recovery process to 6 weeks for complete resolution [9]. This, along with pilot experiments allowed us to determine the 4 weeks recovery phase as suitable to allow for adequate bone marrow reconstitution and studies on ongoing fibrosis resolution.

Interestingly, reconstitution with BM from VHL^fl/fl^-LysMCre+ mice results in reduced liver collagen content compared to mice reconstituted with wildtype (VHL^fl/fl^-LysMCre -) bone marrow (Figure 1C, 1D and 1E) as assessed by quantitative analysis of the Sirius red-positive area on liver sections (Figure 1D) as well as by determination of the total liver hydroxy-proline content (Figure 1E). Consistent with the notion that ECM degradation itself can contribute to myofibroblast contraction upon fibrosis regression [15, 16], we observe reduced numbers of α-SMA-expressing myofibroblasts after reconstitution with VHL^fl/fl^-LysMCre+ BM (Figure 1F). Taken together, this indicates that boosting the hypoxic response in myeloid cells by deleting VHL accelerates the resolution of fibrosis.

### Deletion of VHL in myeloid cells upon resolution enhances ECM degradation activity and endothelial expression of matrix degrading enzymes

**Figure 2:**
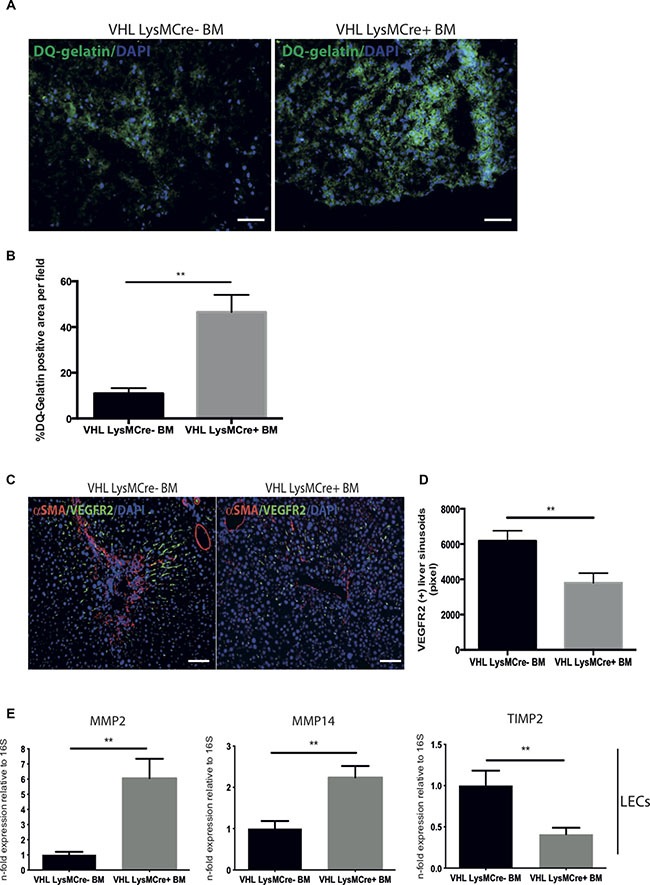
Transplantation of bone marrow from VHL^fl/fl^-LysMcre+ mice into C57Bl6/J mice after CCl_4_-challenge accelerates matrix degradation activity and the expression of matrix degrading enzymes in liver endothelial cells (**A**) DQ™-gelatin images illustrating the gelatinolytic activity in liver sections from the two experimental groups. (**B**) Quantification of DQ™-gelatin-positive areas on liver sections. (**C**) Representative images of murine liver sections co-immunolabeled for VEGFR2 and α-SMA. (**D**) Quantitative analysis of the VEGFR2-positive area. (**E**) Quantitative real time-analysis of MMP2, MMP14, and TIMP2-expression, respectively, in liver endothelial cells (LECs). Error bars represent SEM (*n* = 5 for VHL LysMCre- and *n* = 7 for VHL LysMCre+). Scale bars equal 100 μm.

The resolution of fibrosis requires the breakdown of the ECM network. Hence, we performed an *in situ* zymography by incubating liver sections with fluorescein-labeled gelatin (DQ-gelatin^™^). This fluorogenic substrate yields a bright fluorescent signal upon proteolytic digestion and allows the *in situ* detection of ECM degradation (Figure 2A). Quantitative analysis of the fluorescent signal revealed increased zymographic activity in mice with BM from VHL^fl/fl^-LysMCre+ mice compared to mice after reconstitution with wildtype (VHL^fl/fl^-LysMCre-) bone marrow (Figure 2B). This further suggests that mice reconstituted with BM from VHL^fl/fl^-LysMCre+ mice are more efficient in breaking down ECM and resolving liver fibrosis. We have previously shown that, despite an overall increase in vascular density, the fibrotic scar is mostly devoid of sinusoids, suggesting sinusoidal rarefication in this area [9]. Upon regression of the fibrotic scar, though, the fibrotic areas become revascularized in a VEGF-dependent manner, resulting in a more homogenous distribution of sinusoidal vessels and a decrease in vascular density [9, 17]. This was linked to a proresolution phenotype of the liver endothelium, involving increased expression of MMP-2 and -14 as well as reduced expression of TIMP-1 and -2 in response to myeloid cell-derived VEGF[9]. In order to determine whether targeting of VHL in myeloid cells translates into vascular changes, we performed simultaneous detection of sinusoidal vessels and the fibrotic scar by means of double immunofluorescence for VEGFR2 and SMA on liver sections from both genotypes. As shown in Figure 2C, accelerated resolution of the fibrotic scar in VHL^fl/fl^-LysMCre+ BM-reconstituted mice was indeed associated with a more homogenous pattern of sinusoids and a reduction of vascular density (Figure 2D). Strikingly, this was associated with enhanced expression of MMP-2 and -14 and a decrease in TIMP-2 expression in sorted liver endothelial cells (Figure 2E), thus further substantiating the role of VEGF as a driver of fibrolysis.

### Targeting of VHL in myeloid cells increases macrophage MMP-13 expression

**Figure 3:**
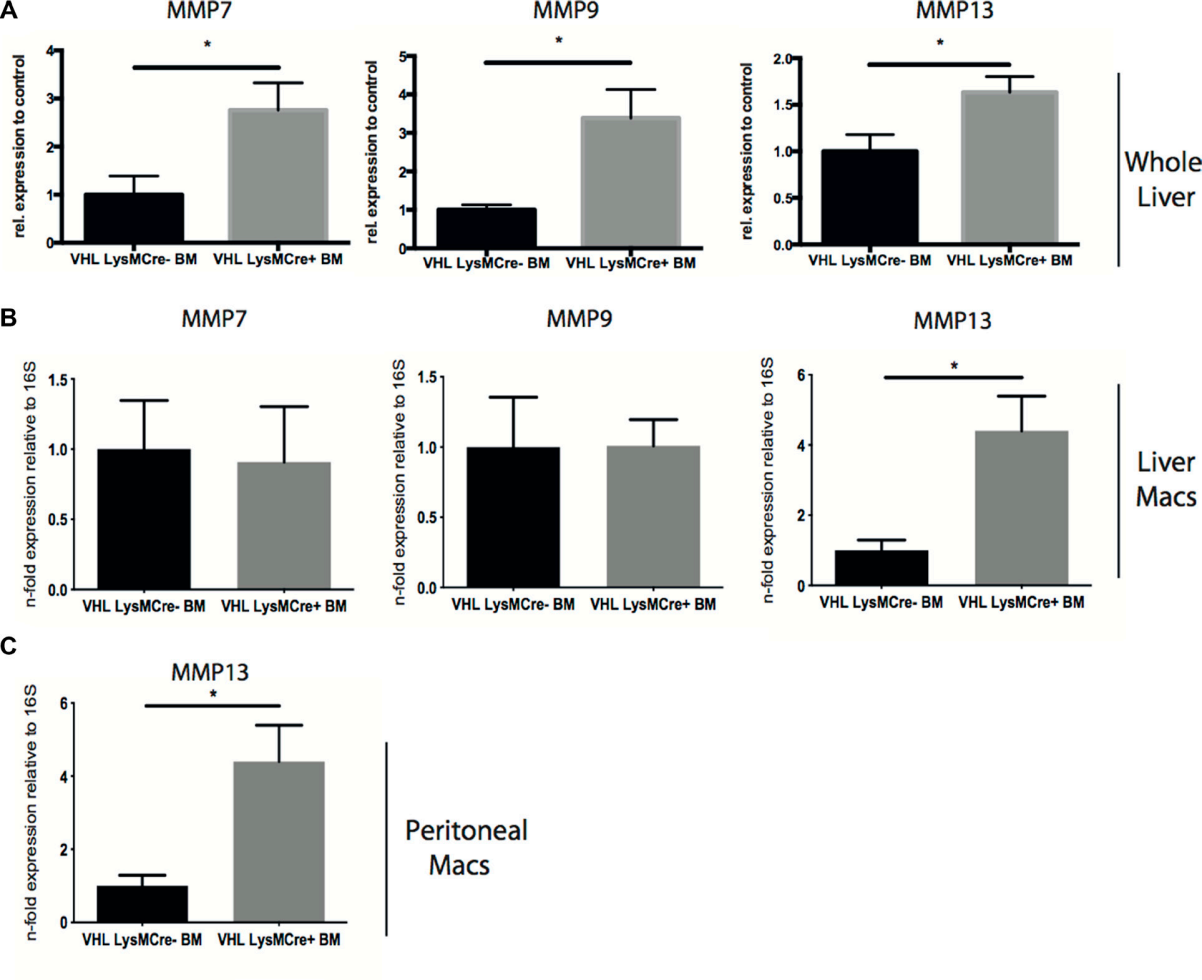
Deletion of VHL in myeloid cells during the resolution of liver fibrosis induces the expression of matrix degrading enzymes in whole liver and liver macrophages (**A**) Quantitative real time-analysis of MMP7, MMP9, and MMP13 expression, respectively, in whole livers (*n* = 5 for VHL LysMCre- and *n* = 7 for VHL LysMCre+). (**B**) Quantitative real time-analysis of MMP7, MMP9, and MMP13 expression, respectively, in isolated liver macrophages (*n* = 5 for VHL LysMCre- and *n* = 7 for VHL LysMCre+). (**C**) Quantitative real time-analysis of MMP13 expression in peritoneal macrophages (*n* = 5 for VHL LysMCre- and *n* = 6 for VHL LysMCre+). Error bars represent SEM.

Furthermore, we observe increased expression of MMP-7, -9 and -13 in whole livers after reconstitution with VHLfl/fl-LysMCre + bone marrow (Figure 3A). Scar associated macrophages have been shown to be a potent source of MMPs and particularly MMP-13 [3, 6]. Consistently, isolated F4-80-positive macrophages from fibrotic livers showed upregulation of MMP-13 expression upon VHL deletion (Figure 3B), whereas MMP-7 and -9 expression in isolated liver macrophages remained similar across genotypes (Figure 3B), pointing towards another, non-macrophage source for these MMPs in our particular setting. Consistently, peritoneal macrophages isolated from VHLfl/fl-LysMCre+ mice also show increased levels of MMP-13 transcripts (Figure 3C). Taken together, this suggests that targeting the hypoxic response in myeloid cells may contribute to the resolution of fibrosis in a much broader sense and not only through VEGF-dependent effects on the liver vasculature.

In addition to macrophages, dendritic cells, Natural Killer (NK) cells and neutrophils have been shown to participate in the regression of liver fibrosis [18–20]. Flow cytometry analysis (Supplementary Figure 1) of fibrotic livers at endpoint revealed that the number of MHCII^+^/CD11C^+^ dendritic cells (Supplementary Figure 2A), NKp46^+^/NK1.1^+^ NK cells (Supplementary Figure 2B) and CD11b^+^/Ly6G^+^ neutrophils (Supplementary Figure 2C) were similar across genotypes. However, reconstitution with VHL^fl/fl^-LysMCre+ BM resulted in decreased numbers of F4/80-expressing macrophages (Supplementary Figure 2D), possibly as a consequence of overall decreased fibrosis at endpoint.

### Deletion of VHL in myeloid cells accelerates liver regeneration

**Figure 4:**
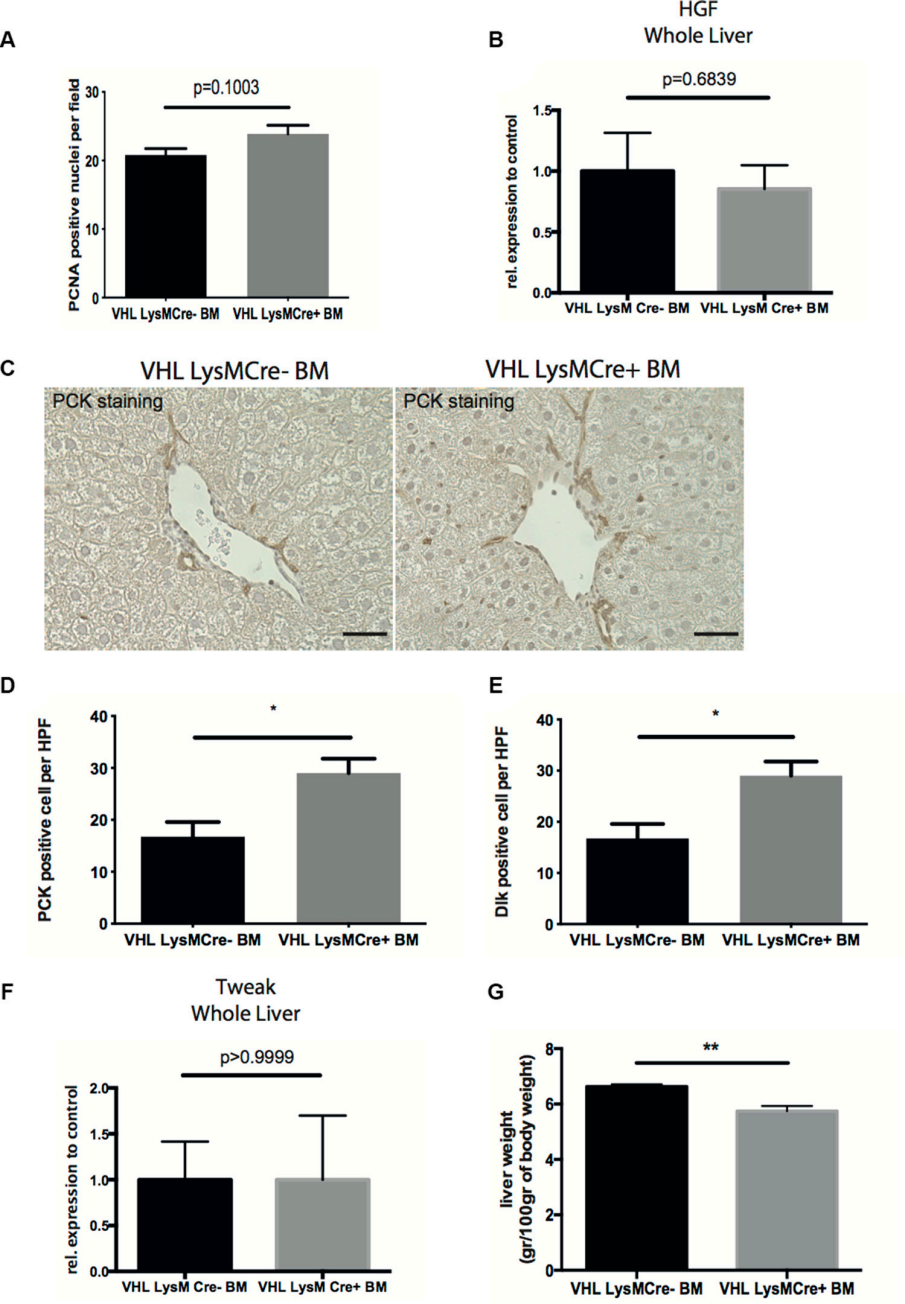
Deletion of VHL in myeloid cells accelerates liver regeneration (**A**) Quantification of PCNA-positive nuclei per field on liver sections. (**B**) Quantitative real time-analysis of HGF expression in whole livers. (**C**) Representative images of pan-cytokeratin (PCK) immunohistochemistry on liver sections. (**D**) Quantitative analysis of PCK-expressing liver progenitor cells. (**E**) Quantitative analysis of Dlk-expressing liver progenitor cells. (**F**) Quantitative real time-analysis of Tweak-expression, respectively, in whole livers. (**G**) Liver wet weight normalized to body weight. Error bars represent SEM (*n* = 5 for VHL LysMCre- and *n* = 7 for VHL LysMCre+). Scale bars equal 50 μm.

Recovery from chronic liver injury also requires regeneration of the liver parenchyma involving the proliferation of hepatocytes as well as the activation of liver progenitor cells [4, 6]. VEGF has been implicated in hepatocyte proliferation and liver regeneration [21, 22]. However, analyzing the number of PCNA-positive proliferating hepatocytes did not reveal differences between genotypes (Figure 4A). Likewise, expression of the hepatocyte mitogen Hepatocyte Growth Factor (HGF) in whole livers remained unchanged (Figure 4B).

Expression of the cytokine TWEAK (tumor necrosis factor-like weak inducer of apoptosis), along with expansion of resident liver progenitor cells has recently been shown to contribute to liver regeneration after CCl4-challenge [6]. Therefore, we wanted to investigate the effect of VHL deletion in myeloid cells on liver progenitor cell compartment. Interestingly, reconstitution with VHL^fl/fl^-LysMCre+ bone marrow results in increased numbers of pancytokeratin- and Dlk-expressing liver progenitor cells, indicating improved liver regeneration along with accelerated fibrolysis (Figures 4C, 4D and 4E). However, this was not associated with differences in whole liver TWEAK expression between genotypes (Figure 4F). Consistent with the notion that resolution of fibrosis and liver regeneration is associated with a reduction of liver weight [23], we observe decreased liver wet weights after reconstitution with VHLfl/fl-LysMCre+ bone marrow (Figure 4G).

Given the current efforts of macrophage-based therapy for liver fibrosis, we propose that enhancing the hypoxic response in scar infiltrating macrophages could represent a therapeutic avenue for the treatment of liver fibrosis and improvement of liver regeneration.

### The effects of VHL deletion in myeloid cells depend largely on VEGF expression

**Figure 5:**
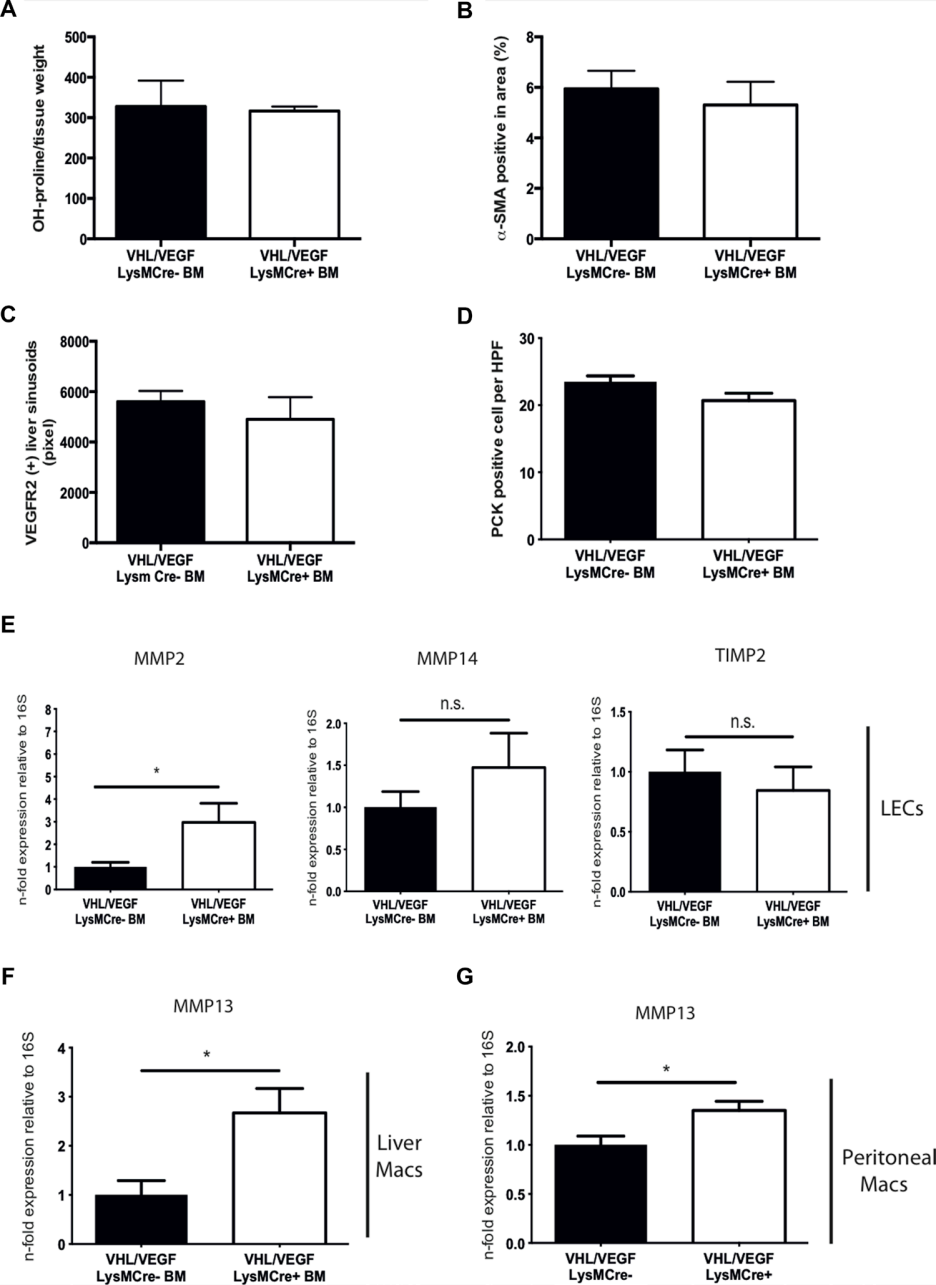
Simultaneous targeting of VEGF and VHL in myeloid cells reverses the phenotype of VHL deletion (**A**) Determination of free hydroxyproline in liver tissue samples (*n* = 5). (**B**) Quantitative analysis of the α-SMA-positive area on liver sections (*n* = 5). (**C**) Quantitative analysis of the VEGFR2-positive area (*n* = 5). (**D**) Quantitative analysis of PCK-expressing liver progenitor cells (*n* =5). (**E**) Quantitative real time-analysis of MMP2, MMP14, and TIMP2-expression, respectively, in liver endothelial cells (LECs) (*n* =5). (**F**) Quantitative real time-analysis of MMP13 expression in isolated liver macrophages (*n* = 5). (**G**) Quantitative real time-analysis of MMP13 expression in peritoneal macrophages (*n* = 5 for VEGF/VHL LysMCre- and *n* = 8 for VEGF/VHL LysMCre+). Error bars represent SEM. Scale bars equal 100 μm.

Ablation of VHL results in constitutive activation of HIF and expression of HIF target genes other than VEGF. Therefore additional, VEGF-independent but HIF-dependent effects may contribute to enhanced fibrolysis in our study. In order to dissect out VEGF-dependent from VEGF-independent effects upon VHL inactivation in myeloid cells, we generated mice with a simultaneous deletion of VHL and VEGF in myeloid cells (VHL^fl/fl^/VEGF^fl/fl^-LysMCre+) and transplanted their BM along with the appropriate controls (VHL^fl/fl^/VEGF^fl/fl^-LysMCre-) into CCl4-treated C57Bl6/J mice after whole body irradiation (10 Gy) (in analogy to Figure 1B), followed by a 4 week recovery phase. As shown in Figure 5, reconstitution with BM from VHL^fl/fl^/VEGF^fl/fl^-LysMCre+ mice results in liver collagen contents (Figure 5A) and numbers of α-SMA-expressing myofibroblasts (Figure 5B) that are as high as in mice reconstituted with wildtype (VHL^fl/fl^/VEGF^fl/fl^-LysMCre-) BM. In addition, the density of VEGFR2 (+) liver sinusoids (Figure 5C) as well as the number of endogenous liver progenitor cells (Figure 5D) are similar across genotypes. Therefore, simultaneous deletion of VEGF and VHL in myeloid cells reverses the phenotype observed after VHL^fl/fl^-LysMCre+ BM transplantation (Figures 1, 2, 3 and 4), indicating that the improved outcome upon VHL deletion in myeloid cells depends largely on increased VEGF expression. Consistently, the double knockout of VHL and VEGF in myeloid cells partially prevents the induction of a proresolution phenotype in the liver endothelium as illustrated by similar expression of MMP-14 and TIMP-2 in sorted liver endothelial cells (Figure 5E). Although, the expression of MMP-2 in the liver endothelium was still significantly increased in mice reconstituted with VHL^fl/fl^/VEGF^fl/fl^-LysMCre+ (Figure 5E), it was lower than in VHL^fl/fl^-LysMCre+ BM reconstitution setting (Figure 2E). Noteworthy, MMP-13 expression in isolated F4/80-positive macrophages from fibrotic livers of VHLfl/fl/VEGFfl/fl-LysMCre+ BM-reconstituted mice (Figure 5F) as well as in VHL/VEGF-deficient peritoneal macrophages (Figure 5G) was lower than in the VHLfl/fl-LysMCre+ setting (compare with Figure 3B and 3C), yet still significantly higher than under wildtype (VHLfl/fl/VEGFfl/fl-LysMCre-) conditions (Figure 5F and 5G, respectively). This indicates that upon VHL deletion in myeloid cells, the proresolution phenotype in the liver endothelium largely depends on VEGF, whereas increased MMP-13 expression in scar associated macrophages seems to be rather VEGF-independent but HIF-dependent.

### Therapy with VHL-deficient macrophages enhances fibrolysis

**Figure 6:**
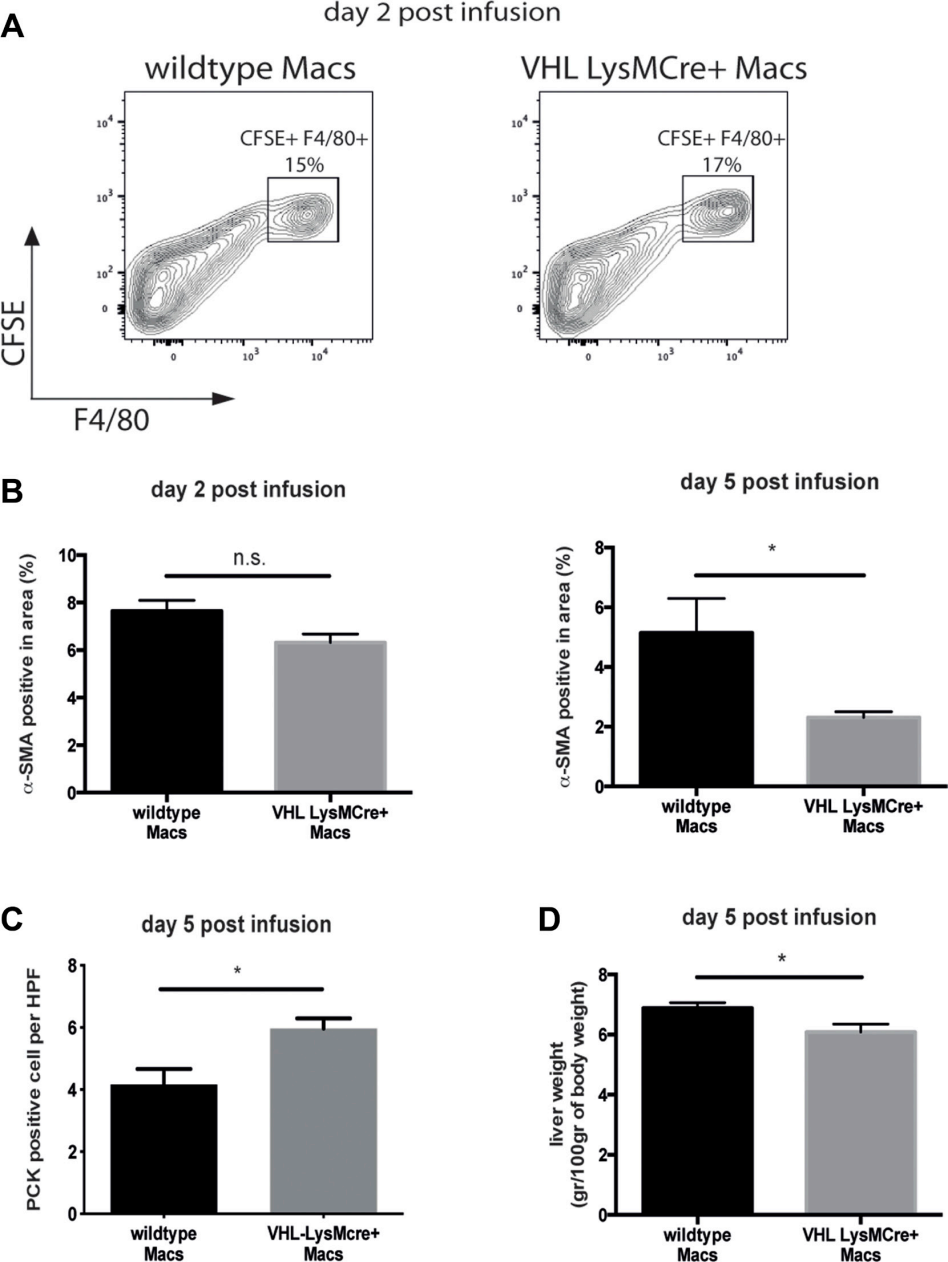
Therapy with VHL-deficient macrophages accelerates fibrosis resolution (**A**) Flow cytometric analysis of F4/80 and CFSE double-positive macrophages on fibrotic livers 2 days after macrophage infusion (*n* = 4). (**B**) Quantitative analysis of the α-SMA-positive area on liver sections (*n* = 5 for VHL LysMCre- and *n* = 6 for VHL LysMCre+). (**C**) Quantitative analysis of PCK-expressing liver progenitor cells (*n* =5 for VHL LysMCre- and *n* = 5 for VHL LysMCre+). (**D**) Liver wet weight normalized to body weight (*n* = 5 for VHL LysMCre- and *n* = 6 for VHL LysMCre+). Error bars represent SEM.

Macrophage infusion is an emerging immunotherapeutic tool for the treatment of liver fibrosis. In murine models, the transfer of F4/80-positive bone marrow-derived, but otherwise unmanipulated macrophages, has been shown to foster scar resolution as well as liver regeneration [6]. Given the superior outcome on fibrolysis and concomitant regeneration of fibrotic livers after reconstitution with VHLfl/fl-LysMCre+ BM along with increased expression of VEGF and MMP-13 in VHL-deficient peritoneal macrophages, we wanted to evaluate the therapeutic potential of boosting the hypoxic response in a setting of macrophage therapy. In order to render our study comparable, we adopted a previously published model of macrophage therapy involving 6 weeks of CCL4-treatment that induces a significant degree of fibrosis compared to sham-treated mice (Supplementary Figure 3A and 3B), followed by infusion of F4/80-positive macrophages [6, 7]. To this end, we isolated thioglycollate-elicited peritoneal macrophages from wildtype (VHLfl/fl-LysMCre-) and VHLfl/fl-LysMCre+ mice, which yielded a CD11b- and F4/80-positive macrophage population regardless of the genotype (Supplementary Figure 3C). After fluorescent labelling with carboxyfluorescein succinimidyl ester (CFSE) (Supplementary Figure 3D), 1×106 macrophages of each genotype were injected intravenously into CCl4-treated C57Bl6/J mice and livers were analyzed at day 2 and 5 post-infusion [7]. As shown in Figure 6A, the transferred, CFSE-labelled macrophages incorporate into fibrotic livers and are present at day 2 post-infusion to similar extent across genotypes.

It has been shown that the success of macrophage therapy depends on additional recruitment of host immune cells [6] as well as the presence of CD11Bhigh F4/80int Ly6Clo “restorative” macrophages with crucial proresolution properties. Flow cytometry analysis of fibrotic livers at day 2 and 5 post-infusion revealed that the number of MHCII^+^/CD11C^+^ dendritic cells (Supplementary Figure 4A), NKp46^+^/NK1.1^+^ NK cells (Supplementary Figure 4B) and CD11b^+^/Ly6G^+^ neutrophils (Supplementary Figure 4C) and F4/80-expressing macrophages (Supplementary Figure 4D) were similar across genotypes. Moreover, we confirm the CD11Bhigh F4/80int Ly6Clo restorative macrophage as the predominant cell type during early fibrolysis (Supplementary Figure 4E). Yet, the number of CD11Bhigh F4/80int Ly6Clo restorative macrophages was similar across genotypes (Supplementary Figure 4E). However, we observe reduced numbers of α-SMA-expressing myofibroblasts and subtle but significantly increased expansion of endogenous liver progenitor cells along with decreased liver weights at day 5 after therapy with VHL-deficient macrophages (Figure 6B, 6C and 6D, respectively). This indicates that forced VEGF expression within the CD11B_high_ F4/80_int_ Ly6C_lo_ population of restorative macrophages could drive improved scar resolution and liver regeneration in this setting.

### Induction of the hypoxic response in infused macrophages by prolyl hydroxylase inhibition accelerates fibrosis resolution and liver regeneration

**Figure 7:**
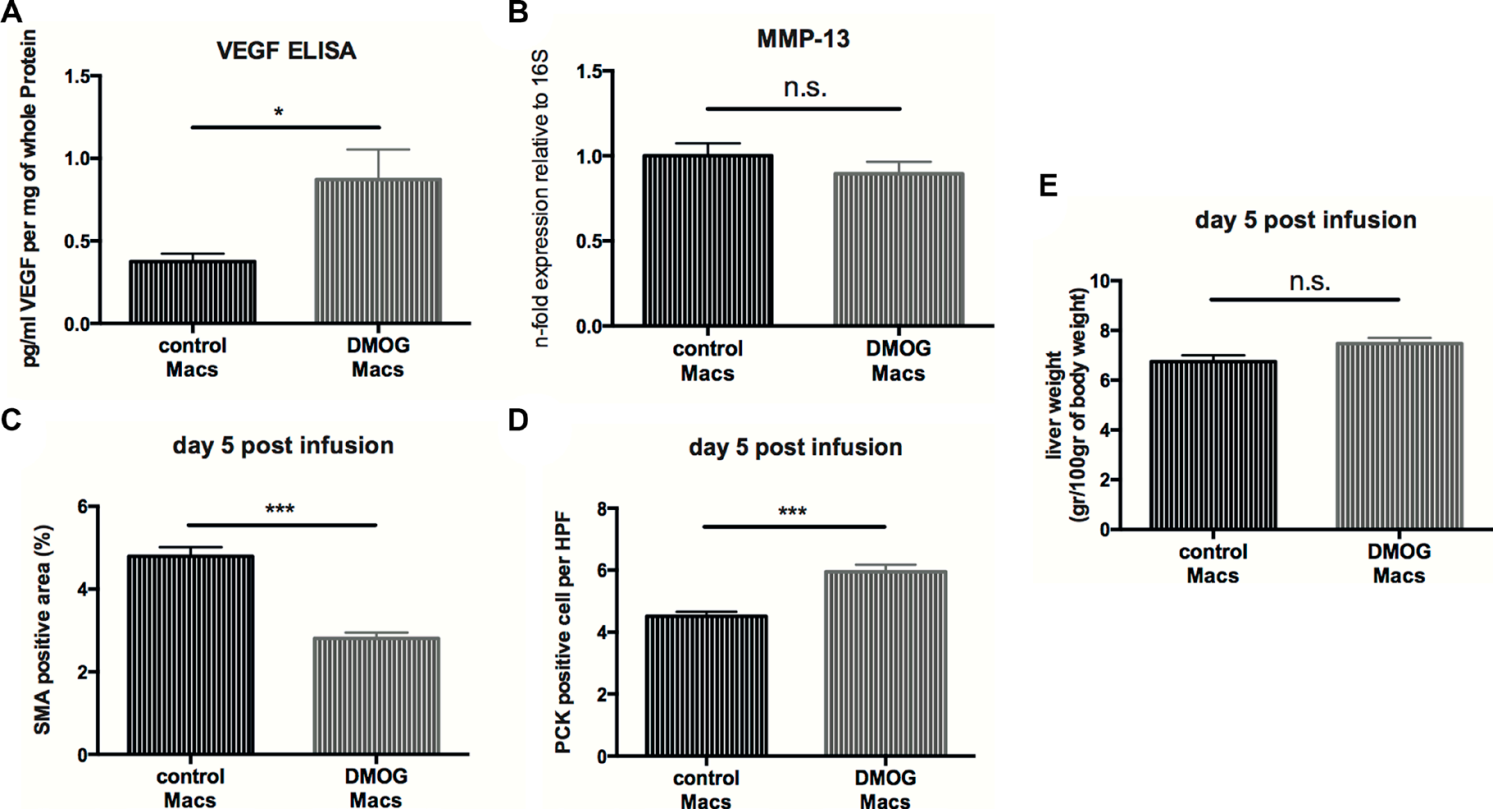
Infusion of macrophages treated with a HIF-inducing prolyl-hydroxylase inhibitor accelerates fibrosis resolution (**A**) VEGF release by peritoneal macrophages (Macs) treated with 1mM DMOG in DMSO or control vehicle for 12 hours (*n* = 10). (**B**) Quantitative real time-analysis of MMP13 expression in peritoneal macrophages treated with 1mM DMOG in DMSO or control vehicle for 12 hours (*n* =10). (**C**) Quantitative analysis of the α-SMA-positive area on fibrotic livers 5 days after infusion of macrophages treated with 1mM DMOG in DMSO or control vehicle (*n* = 7). (**D**) Quantitative analysis of PCK-expressing liver progenitor cells on fibrotic livers 5 days after infusion of macrophages treated with 1mM DMOG in DMSO or control vehicle (*n* = 7). (**E**) Wet weight normalized to body weight of fibrotic livers 5 days after infusion of macrophages treated with 1mM DMOG in DMSO or control vehicle (*n* = 7). Error bars represent SEM.

Small-molecule stabilizers of hypoxia inducible factor (HIF) have been developed for the treatment of various diseases. These molecules inhibit prolyl hydroxylase domain-containing (PHD) enzymes, resulting in constitutive HIF activation and some PHD inhibitors are currently in clinical trials [24]. Therefore, we wanted to evaluate the therapeutic potential of this class of compounds in a setting of macrophage therapy and treated peritoneal macrophages with the prolyl-hydroxylase inhibitor and HIF-inducer Dimethyloxalylglycine (DMOG) or control vehicle prior to infusion into CCL4-treated mice. *Ex vivo* analysis peritoneal macrophages shows increased VEGF expression upon DMOG-treatment (Figure 7A) whereas levels of MMP-13 transcripts remained unchanged (Figure 7B). Next, 1 × 10^6^ macrophages treated with DMOG or control vehicle were injected intravenously into CCl4-treated C57Bl6/J mice and livers were analyzed at day 5 post-infusion in analogy to our above-mentioned experimental setup.

Flow cytometry analysis of fibrotic livers at day 5 post-infusion revealed that the number of MHCII^+^/CD11C^+^ dendritic cells (Supplementary Figure 5A), NKp46^+^/NK1.1^+^ NK cells (Supplementary Figure 5B) and CD11b^+^/Ly6G^+^ neutrophils (Supplementary Figure 5C) were similar across treatment modalities. However, a slight increase in F4/80-expressing macrophages after DMOG-treatment (Supplementary Figure 5D), whereas the number of CD11Bhigh F4/80int Ly6Clo restorative macrophages was similar across genotypes (Supplementary Figure 5E). Yet, we observe reduced numbers of α-SMA-expressing myofibroblasts (Figure 7C) and a significantly increased expansion of endogenous liver progenitor cells (Figure 7D). However, infusion of DMOG-treated macrophages did not result in decreased liver weights at day 5 after therapy (Figure 7E).

Taken together, this indicates that boosting the hypoxic response in macrophages exogenously may be effective in macrophage therapy for liver fibrosis.

## DISCUSSION

Mouse studies have shown that transfer of untreated cells of the monocyte-macrophage lineage is able to reduce fibrosis as well to foster liver regeneration [6] and clinical testing of this approach is on the way [4]. We have previously shown that sinusoidal angiogenesis driven by myeloid cell-derived VEGF along with upregulation of MMP-2 and MMP-14 in sinusoidal endothelial cells is required for the resolution of liver fibrosis [9]. Here we show that the resolution of fibrosis can be accelerated by deleting VHL, a negative regulator of HIFs and VEGF, specifically in myeloid cells. Consistently, this was associated with increased VEGF expression in macrophages, increased expression of MMP-2 and MMP-14 but lower transcripts of TIMP-2 in sinusoidal endothelial cells upon recovery, resulting in enhanced ECM degradation and loss of liver myofibroblasts. In addition, this was associated with increased whole liver expression of MMP-7, and -9, and upregulation of MMP-13 expression in isolated liver macrophages.

Importantly, ablation of VHL results in constitutive activation of HIF and expression of HIF target genes other than VEGF. Therefore additional, VEGF-independent but HIF-dependent effects may contribute to enhanced fibrolysis in our study. Indeed, in the context of liver fibrosis, myeloid cells, including macrophages, neutrophils and dendritic cells are important sources of MMP-13 and MMP-9 [3, 6, 18], respectively. Noteworthy, both MMPs are considered to be HIF targets [25]. Consistent with this, we observe increased expression of MMP-9 and MMP-13 in whole livers from mice reconstituted with VHL^fl/fl^-LysMCre+ BM. Therefore, enhanced expression of MMP-9 and MMP-13 upon deletion of VHL specifically in myeloid cells including could contribute the accelerated resolution of fibrosis in our study. However, in isolated liver macrophages, only the expression of MMP-13 was elevated upon deletion of VHL, suggesting neutrophils or dendritic cells as a possible alternative MMP-9 source [3, 6, 18] in our setting. Noteworthy, treatment of peritoneal macrophages with the HIF-inducing prolyl hydroxylase inhibitor DMOG that increases VEGF- but not MMP-13 expression, still leads to accelerated fibrolysis upon infusion.

We observe reduced numbers of α-SMA-expressing myofibroblasts in livers from mice reconstituted with VHL^fl/fl^-LysMCre+ BM as well as after infusion of VHL null or DMOG-treated macrophages. This is consistent with the notion that ECM degradation itself can induce myofibroblast contraction upon fibrosis regression[15, 16]. Yet, it remains to be determined whether this is due to increased apoptosis/inactivation or reduced proliferation of myofibroblasts.

Noteworthy, the major phenotype of myeloid-specific VHL inactivation, including accelerated fibrosis resolution and improved liver regeneration is reversed upon additional deletion of VEGF in myeloid cells. This strongly suggests that indeed VEGF-dependent effects are largely responsible for the enhanced outcome. Yet, the expression of MMP-2 in the liver endothelium, although lower than in VHL^fl/fl^-LysMCre+ setting, was still significantly increased in mice reconstituted with VHL^fl/fl^/VEGF^fl/fl^-LysMCre+ BM, presumably due to the action of angiogenic factors other than VEGF. Likewise, simultaneous deletion of VHL and VEGF does not entirely suppress increased macrophage MMP-13 expression. This suggests, first, that autocrine VEGF could partially regulate MMP-13 expression in macrophages and second VEGF-independent but indeed HIF-dependent mechanism of MMP-13 expression in myeloid cells upon VHL deletion. However, these subtle increases in MMP-2 and MMP-13 expression after reconstitution with VHL^fl/fl^/VEGF^fl/fl^-LysMCre+ BM, although statistically significant, are obviously not sufficient to accelerate the resolution of liver fibrosis. Along with this, exposure of peritoneal macrophages to the prolyl hydroxylase inhibitor DMOG only increases VEGF- and not MMP-13 expression, yet still leads to accelerated fibrolysis in a setting of macrophage therapy.

A recent mouse study showed that infusion and transient engraftment of BM-derived macrophages improves fibrosis resolution by recruiting additional endogenous immune cells as well as by paracrine induction of a fibrolytic phenotype in those host-derived cells [6]. In addition, deletion of VHL in myeloid cells has been reported to enhance immune cell recruitment [14]. In our study, although we do not observe enhanced immune cell recruitment, the expression of MMP-9 and MMP-13 is increased after reconstitution with VHLfl/fl-LysMCre+ bone marrow. This further suggests that VHL deletion in the myeloid cell compartment promotes fibrosis regression by inducing a VEGF-expressing proresolution myeloid cell phenotype rather than by enhancing recruitment of additional immune cells. Interestingly, pretreatment of macrophages with DMOG prior to therapeutic infusion results in higher numbers of F4/80-expressing macrophages along with increased fibrosis resolution. However, it remains to be determined whether this is due to improved survival of transferred macrophages or enhanced recruitment of endogenous macrophage populations.

In this context, antifibrotic, “restorative” macrophages with a CD11B^high^ F4/80^int^ Ly6C^lo^ phenotype outside the M1/M2 macrophage nomenclatura and with exquisite proresolution properties haven been shown to be crucial during early scar resolution[7]. In our setting with therapeutic infusion of VHL-deficient macrophages that results in improved scar resolution, the number of cells with CD11B^high^ F4/80^int^ Ly6C^lo^ phenotype is similar across genotypes. Yet, our experimental setup confirms CD11B^high^ F4/80^int^ Ly6C^lo^ restorative macrophage as the predominant cell type during early fibrolysis. This allows the intriguing hypothesis that preconditioning of the hypoxic response within the CD11B^high^ F4/80^int^ Ly6C^lo^macrophage population could further enhance proresolution properties upon therapeutic infusion without affecting the surface marker expression profile of this crucial cell type. Moreover, the two major isoforms HIF-1 and HIF-2 have been shown to play an important role M1/M2 macrophage polarization [26]. Hence, despite the fact that M1/M2 characterization does not accurately reflect the macrophage phenotypes observed the context of liver fibrosis [6, 7], it will be important to dissect out the presumably non-overlapping roles of HIF-1 and HIF-2 in myeloid cells during fibrosis resolution. For instance, HIF-1 has been shown to be essential for macrophage phagocytosis [14]. Therefore, constitutive activation of HIF-1 in myeloid cells in the absence of VHL or after DMOG treatment could enhance phagocytic activity and thereby contribute to fibrosis regression.

Finally, it is important to mention that at least in tumor cells a functional VHL pathway is involved in assembly and remodeling of the ECM (e.g collagen IV and fibronectin) in a HIF-independent manner [27–29]. However, it remains to be elucidated whether these mechanisms can be applied to the myeloid cell compartment.

In addition to accelerated fibrosis resolution we observe improved liver regeneration in mice reconstituted VHL^fl/fl^-LysMCre+ bone marrow which was abrogated by additional deletion of VEGF. VEGF has been implicated in early liver regeneration by stimulating hepatocyte proliferation, either directly or in an angiocrine manner [21, 22] e.g., by stimulating endothelial HGF release. Therefore, increased myeloid cell VEGF expression upon VHL deletion or DMOG treatment could foster hepatocyte proliferation. Analyzing the number of PCNA-positive proliferating hepatocytes as well as hepatic HGF expression did not reveal differences between genotypes. Taken together, this argues against a crucial role of myeloid cell-derived VEGF in hepatocyte proliferation.

However, recently Thomas et al. [6] successfully used macrophage therapy to improve liver regeneration. In this study, parenchymal regeneration was entirely driven by liver progenitor cell expansion and this was associated with increased expression of VEGF and the cytokine TWEAK[6]. Similarly, we do observe significant expansion of liver progenitor cells in mice reconstituted VHL^fl/fl^-LysMCre+ bone marrow, although, in the absence of increased TWEAK expression, that is again lost upon reconstitution with VHL^fl/fl^/VEGF^fl/fl^-LysMCre+ BM. Although, not analyzed in the study, it is important to mention that Wnt3a expression in macrophages has been shown to drive progenitor cell expansion and liver regeneration in a paracrine fashion [30]. Alternatively, it has been shown that (human) hepatic progenitors express VEGFR1, opening up a possible direct role for VEGF in liver progenitor expansion. Finally, scar degradation by itself can facilitate progenitor cell expansion [31]. Thus, it is likely that accelerated scar resolution and ECM remodeling as observed in mice reconstituted VHL^fl/fl^-LysMCre+ bone marrow as well as upon infusion of VHL-deficient or DMOG-treated macrophages indirectly promotes the process of liver regeneration as reflected by increased number of liver progenitor cells and reduced liver weight. Yet, it remains to be determined whether this translates into improved liver function.

Autologous cell therapy is an emerging immunotherapeutic tool in the context of chronic liver disease. Our results suggest that manipulating the HIF-VEGF signaling axis prior to transfer could further enhance immunotherapeutic outcome. Indeed, infusion of VHL-deficient or DMOG-treated macrophages that overexpress VEGF and/or MMP-13 reduces α-SMA myofibroblasts numbers and increases expansion of endogenous liver progenitor cells in fibrotic livers. Yet, potential deleterious effects of augmented HIF-VEGF signaling in precancerous fibrosis/cirrhosis with respect to malignant progression have to be taken in account.

Taken together, our results indicate that broad activation of the hypoxic response by prolyl hydroxylase inhibitors in myeloid cells along with augmented VEGF expression upon recovery is favorable with regard to scar resolution and liver regeneration. This has potential implication for the use of HIF-inducing prolyl hydroxylase inhibitors in the setting of autologous cell therapy, e.g. *ex vivo* treatment of cells of the monocyte/macrophage lineage with such compounds prior to transfer. The data reported here could inform the design of clinical studies involving HIF-inducing prolyl hydroxylase inhibitors to improve the efficacy of autologous cell therapy in chronic liver disease.

## MATERIALS AND METHODS

### Animals

The Animal Care and Use Committee of the Bezirksregierung Düsseldorf, Germany, approved all procedures performed on mice. Myeloid cell-specific knock out of VHL was achieved by breeding male Mice (C57Bl/6), with both alleles of VHL flanked by loxP sites (VHL+^f^/+^f^) with female mice (C57Bl/6) homozygous for the floxed VHL allele expressing Cre recombinase driven by the lysozyme M promoter (VHL+^f^/+^f^-LysMCre+)[17]. Simultaneous knock out of VHL and VEGF in myeloid cells was achieved by breeding male Mice (C57Bl/6), with both alleles of VHL and VEGF flanked by loxP sites (VHL^fl/fl^/VEGF^fl/fl^) with female mice (C57Bl/6) homozygous for the floxed VHL and VEGF alleles expressing Cre recombinase driven by the lysozyme M promoter (VHL^fl/fl^/VEGF^fl/fl^-LysMCre+) [9, 17]. As bone marrow donor mice we used mice carrying the floxed alleles and positive for cre-expression (VHL+^f^/+^f^-LysMCre+) and VHL^fl/fl^/VEGF^fl/fl^-LysMCre+, respectively) and female littermates negative for cre expression served as wildtype controls. All animals received care according to the “Guide for the care and use of laboratory animals”. The number of animals per experimental group to achieve a minimal effect of 25% with a power of 80% was calculated based on previous studies [6, 7, 9] with 10 to 14 (depending on the experimental setup). According to the guidelines of the Animal Care and Use Committee of the Bezirksregierung Düsseldorf, Germany, the study was started 5–7 animals per group (depending on the experimental setup) followed by an interim analysis. In case, the observed effects were greater than initially expected and the results reached the level of statistical significance (*p* < 0,05), the experiment was terminated. In case the results showed a clear trend without reaching statistical significance, the remaining animals were included into the experiment.

### Induction of hepatic fibrosis and tissue preparation

For the induction of hepatic fibrosis female mice were treated with CCl_4_ intraperitoneally (240 μl CCl_4_ suspended in olive oil per kg body weight 3 times a week) for 6 weeks (macrophage therapy experiments as previously reported in [6, 7]) and 12 weeks (bone marrow transplantation experiments as previously reported in[9]), respectively. Control mice received i.p. injections of 100 μl olive oil. Mice were sacrificed at indicated time points and livers harvested for further analysis. For histology, livers were fixed in 4% (w/v) PFA overnight and embedded in paraffin or alternatively frozen in O.C.T. tissue TEK. For RNA and protein isolation livers were separated and snap-frozen in liquid nitrogen.

### Immunofluorescence and immunohistochemistry

5-μm sections were deparaffinized with xylene and rehydrated in a graded ethanol series. Antigen retrieval was performed by boiling the sections in low-pH citrate buffer for 15 minutes. Sections were treated with 3% (v/v) H2O2 for 10 min at RT, blocked with 5% normal goat serum (Sigma) for 1 hour at RT and incubated with the primary antibody overnight at 4°C. Antigens of interest were visualized using the Vectastain ABC kit (Vector Laboratories) or by species-specific fluorochrome-conjugated secondary antibodies. For stainings with mouse-derived antibodies, *Mouse* on *mouse* (*M.O.M*.) Basic *Kit* (Vector Laboratories) was used following the kit instructions. Cell nuclei were stained with DAPI (Invitrogen) and coverslips were mounted with Mounting Medium (Dako). The following antibodies were used in this study: mouse α-SMA at 1:500 dilution (Sigma), rabbit VEGFR-2 at 1:100 dilution (Cell signaling), mouse PCNA (Sigma) at 1:200, PCK (Dako) at 1:200, Dlk (Abcam) at 1:150.

### Sirius red staining

Liver tissues were stained for collagen using the Picrosirius Red Stain kit (Polysciences Inc.) following the manufacturer's instructions. For quantitative analysis of, a minimum of 10 non-overlapping fields of each sections were photographed (Nikon Eclipse E1000 microscope and the Nikon DS-Ri1 camera system) at 200×. The percentage of Sirius red staining was measured with Image J (National Institute of Health).

### *In situ* zymography

A mixture of DQ™-gelatin and reaction buffer (Invitrogen) was applied on top of OCT-embedded frozen sections and incubated at 37°C for 24 h in a dark humid chamber. The gelatinolytic activity was observed as green fluorescence by fluorescence microscopy (excitation: 342 nm; emission: 441 nm).

### Quantification of histology markers

For quantitative analysis of immunohistochemical markers, sections were photographed into JPEG images (Nikon Eclipse E1000 microscope and the Nikon DS-Ri1 camera system). For the quantification of α-SMA and VEGFR-2 the number of pixels marked above a threshold by each marker was measured using the ImageJ program (National Institute of Health) and calculated as the percentage of the total area covered by DAPI. For assessment of DLK, PCK and PCNA, positive cells were counted in each field.

### RNA extraction and qPCR-analysis

Total RNA was isolated by using the Qiagen Midi Kit^®^ according to the manufacturers instructions. cDNA was synthesized from 1 μg of DNA-free total RNA in a 25 μl reaction volume using a Reverse Transcriptase kit (Eurogentec). Gene-specific transcription levels were determined in a 20 μl reaction volume in duplicate using SYBR Green Mastermix (Promega) and an IQ5 real-time PCR machine (Bio-Rad). Quantification was done in a two-step real-time PCR with a denaturation step at 95°C for 10 min and followed by 40 cycles at 95°C for 15 s and at 60 °C for 1 min. Data were normalized by the level of 16S mRNA expression. Primers used in RT-qPCR reactions were as follows:

16S: forward primer: 5′-AGATGATCGAGCCGCGC-3′,

reverse primer: 5′-GCTACCAGGGCCTTTGAGATGGA-3′;

MMP2: GGCTGGAACACTCTCAGGAC-3′,

reverse primer: 5′-CGATGCCATCAAAGACAATG-3′;

MMP14: forward primer: 5′-GTGCCCTATGCCTACATCCG-3′,

reverse primer: 5′-CAGCCACCAAGAAGATGTCA-3′;

TIMP2: forward primer: 5′-GGAATGACATCTATGGCAACC-3′,

reverse primer: 5′-GGCCGTGTAGATAAACTCGAT-3′;

MMP9: forward primer: 5′-CGGCACGCTGGAATGATC-3′,

reverse primer: 5′-TCGAACTTCGACACTGACAAGAA-3′;

MMP7: forward primer: 5′-GCTCTCAGAATGTGG AGTATGC-3′,

reverse primer: 5′-AAGTTCACTCCTGCGTCC-3’;

MMP13: forward primer: 5′-TGATGGCACTGCTGACATCAT-3′,

reverse primer: 5′-TGTAGCCTTTGGAACTGCTT-3′.

Predesigned and validated primer sets for hepatocyte growth factor (HGF) and TNF-like weak inducer of apoptosis (TWEAK) were purchased from Qiagen.

### Bone marrow isolation and adoptive cell transfer

Hind limbs of donor mice were removed and cleaned. Both tops of the femur were cut off and each bone flushed with 5 ml RPMI 1640 containing 2% FBS, 10 units/ml heparin, penicillin and streptomycin. The solution was filtered through a sterile 40 μm cell strainer (BD Bioscience), washed twice and the cells directly used for injection.

1 week before and 2 weeks after irradiation (10 Gray), recipient mice were given acidified water (pH 2.6) supplemented with 10 mg/ml Neomycin and 25 mg/ml Polymyxin B (Sigma). The animals were irradiated 48 hours after the last CCl_4_-injection and adoptive transfer of bone marrow cells was performed 24 hours after irradiation. For adoptive transfer, 5 × 10^6^ cells of the isolated bone marrow were injected into the tail-vain of the recipient mice. The mice were subsequently left for bone marrow reconstitution and recovery for 4 weeks, a time point that had been determined as suitable in pilot experiments.

### Isolation of peritoneal macrophages

Mice were injected with 1 ml of thioglycollate 3% in water and sacrificed 72 hours later. After disinfecting the abdomen with 70% ethanol, the skin of the abdomen was removed to expose the peritoneal cavity. 10ml of cold PBS were injected into the peritoneal cavity using a 20ml syringe with a 21G needle. The abdomen was rubbed softly. Afterwards, the injected PBS was collected and the cell suspension was centrifuged at 1200rpm for 5 minutes at 4°C. Where indicated isolated macrophages were treated with 1mM DMOG in DMSO or control vehicle for 12 hours prior to *ex vivo* analysis.

### Macrophage therapy

Thioglycollate-elicited macrophages were labelled with carboxyfluorescein succinimidyl ester (CFSE) and 1 × 10^6^ cells were injected intravenously in fibrotic mice 24 hours after the last CCl4-injection. For DMOG treatment, isolated macrophages were exposed to 1 mM DMOG in DMSO or control vehicle for 1 hour prior to therapeutic infusion. The animals were sacrificed at the indicated time points for further analysis.

### Isolation of liver endothelial cells and macrophages

For isolation of liver endothelial cells, freshly harvested murine livers were homogenized by mechanical disaggregation and digested in cell lysis buffer (DMEM + 2 mg/ml collagenase type III) for 1 hour at 37°C. Single cell suspensions were generated by passing the cells through a 40-μm cell strainer, followed by resuspension in MACS-buffer according to the manufacturer's instructions (Miltenyi Biotec) and incubation of 4 × 10^7^ cells with mouse CD31- or F4/80-antibodies for 45 min at 4°C. The cell suspensions were washed with MACS-buffer and incubated with secondary IgG microbeads for 15 min at 4°C before positive selection using an automated MACS separator (Miltenyi Biotec). The purity of the cell isolates was tested by immunocytochemistry for VEGFR2 and MAC2 after fixing an aliquot of isolated cells on a slide with 4 % PFA in order to identify endothelial cells and macrophages, respectively. The purity for endothelial cells was 88.52 % ± 2.63 (*n* = 10) and 82.73 % ± 3.47 (*n* = 8) for macrophages.

### Flow cytometry analysis

Hepatic nonparenchymal cells containing the total hepatic leukocyte population were purified using Liver Dissociation Kit (Miltenyi Biotec) and a MidiMACS™ Separator (Miltenyi Biotec) according to the manufacturer's instructions. 10^6^ hepatic nonparenchymal cells were incubated with Fc-Block (BD Biosciences ; 553141 ; 1/100) before labeling with fluorochrome-conjugated antibodies. The following fluorochrome-conjugated antibodies were used: anti-F4/80 (Biolegend; 123107; 1/50); anti-Ly6G (Biolegend; 127608, 1/50); anti-NK1.1 (Biolegend; 108748, 1/100); anti-CD3 (BD Biosciences; 562286, 1/100); anti-CD45 (eBioscience; 45-0451; 1/50); anti-Ly6C (eBioscience; 17-5932; 1/50); anti-CD11b (BD Biosciences, 560455, 1/800); anti-MHC II (eBioscience; 11-5321, 1/50); anti-NK1.1 (BD Biosciences; 553165; 1/50); anti-CD19 (BD Bioscience; 562291, 1/200); anti-CD335 (NKp46; eBioscience; 25-3351; 1/50), anti-CD11c (Biolegend; 117309; 1/50); anti-F4/80 (Biolegend; 123117; 1/50) and anti-MHC II (eBioscience; 48-5321; 1/50). LIVE/DEAD^®^ Fixable Aqua Dead Cell Stain Kit (Thermo Fisher Scientific ; L34957) was used as viability dye. Fix cells using a BD Cytofix/CytopermTM solution (Cat. No. 554722).

The gating strategy is depicted in Supplementary Figure 1. The single cell leukocyte population was selected by FSC-H versus FSC-A and SSC-A versus FSC-A, respectively. The leukocyte population was further analyzed for their uptake of the Live/Dead Aqua stain to determine live versus dead cells and for the expression of CD45. Then CD45+ cells were classified as neutrophils by expression of CD11b and Ly6G, dendritic cells by MHC II and CD11c expression, NK cells by NK1.1 and NKp46 expression and macrophage population by co-expression of F4/80 and CD11b. Labeled cells were analyzed on a LSR II flow cytometer (BD Biosciences) and were evaluated with FlowJo Mac software, version 10.0.8r1.

### Mouse VEGF-A ELISA

Thioglycollate-elicited peritoneal macrophages were cultured for 12 hours. Mouse VEGF-A ELISA on supernatants was performed with Quantikine ELISA, Mouse VEGF Immunoassay, R&D Systems. The results are represented as pg/mL VEGF per mg of whole protein.

### Hydroxyproline-assay

Hepatic hydroxyproline content was quantified colorimetrically using snap frozen liver samples. Tissue (~100 mg) was homogenized in distilled water and protein was precipitated using trichloroacitic acid. Samples were washed with ethanol, dried and hydrolyzed in 6 M HCl at 110°C for 18 hours. The hydrolysate was filtered and neutralized with 10 M NaOH. Samples were then incubated with freshly prepared chloramine T solution. Ehrlich's solution was added and samples incubated for 20 minutes at 65°C. The optical density of each sample and serial dilutions of trans-4-hydroxy-L-proline standard (Sigma, Saint Louis, MO) was measured at 550 nm. Hepatic hydroxyproline content is expressed as μg hydroxyproline per gram liver.

### Statistical analysis

Statistical analysis was done using Prism 6.0 software (GraphPad Software). Statistical significance was determined by unpaired students-*t* test. Data are expressed as mean +/− SEM. Statistical significance is indicated as * p<0.05, **p < 0.01, ***p < 0.001, ****p < 0.0001. *n* = 5 for VHL LysMCre- BM and *n* = 7 for VHL LysMCre+ BM.

## SUPPLEMENTARY MATERIALS FIGURES AND TABLES


