# Supplementary material for: Boosting the hypoxic response in myeloid cells accelerates resolution of fibrosis and regeneration of the liver in mice
Source: Oncotarget. 2017 Jan 19;8(9):15085–100. doi: 10.18632/oncotarget.14749 (PMC5362469; doi:10.18632/oncotarget.14749)
Supplement: Supplementary file 1 [file oncotarget-08-15085-s001.pdf]

Boosting the hypoxic response in myeloid cells accelerates resolution of fibrosis and regeneration of the liver in mice

Supplementary Materials

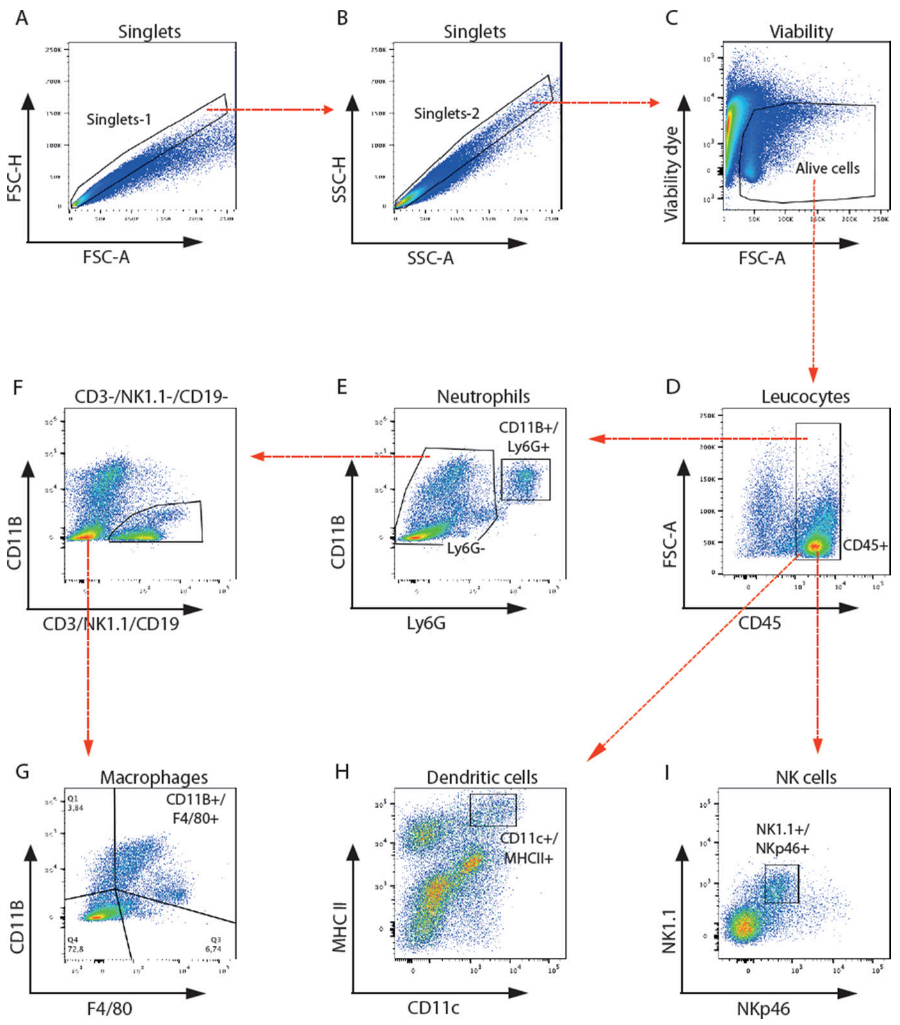

Supplementary Figure 1: Gating strategy for identification of hepatic macrophages, neutrophils, dendritic cells and NK cells. The hepatic nonparenchymal cell fraction was isolated as detailed in Materials and Methods. (**A**) The single cell leukocyte population was selected by FSC-H versus FSC-A and (**B**) by SSC-A versus FSC-A. (**C**) Viable cells were selected by the Live/Dead Aqua stain exclusion. (**D**) CD45-postive viable cells were gated. (**E**) Neutrophils were identified as viable $CD45^{+}$ $CD11B^{+}$ and $Ly\text{-}6G^{+}$ cells, and they were excluded from subsequent macrophage gating. (**F**) Cells positive for CD3, CD19 or NK1.1 were excluded from subsequent macrophage gating. (**G**) Macrophages were selected as viable $CD45^{+}$ $Ly\text{-}6G^{-}$ $CD3^{-}$ $CD19^{-}$ NK1.1- and dual-positive $CD11B^{+}$ $F4/80^{+}$ cells. (**H**) Dendritic cells were selected as viable $CD45^{+}$ MHC II^{+} $CD11c^{+}$ cells. (**I**) Natural Killer cells were selected as viable CD45+ NK1.1+ $NKp46^{+}$ cells. Representative flow cytometry plots are shown.

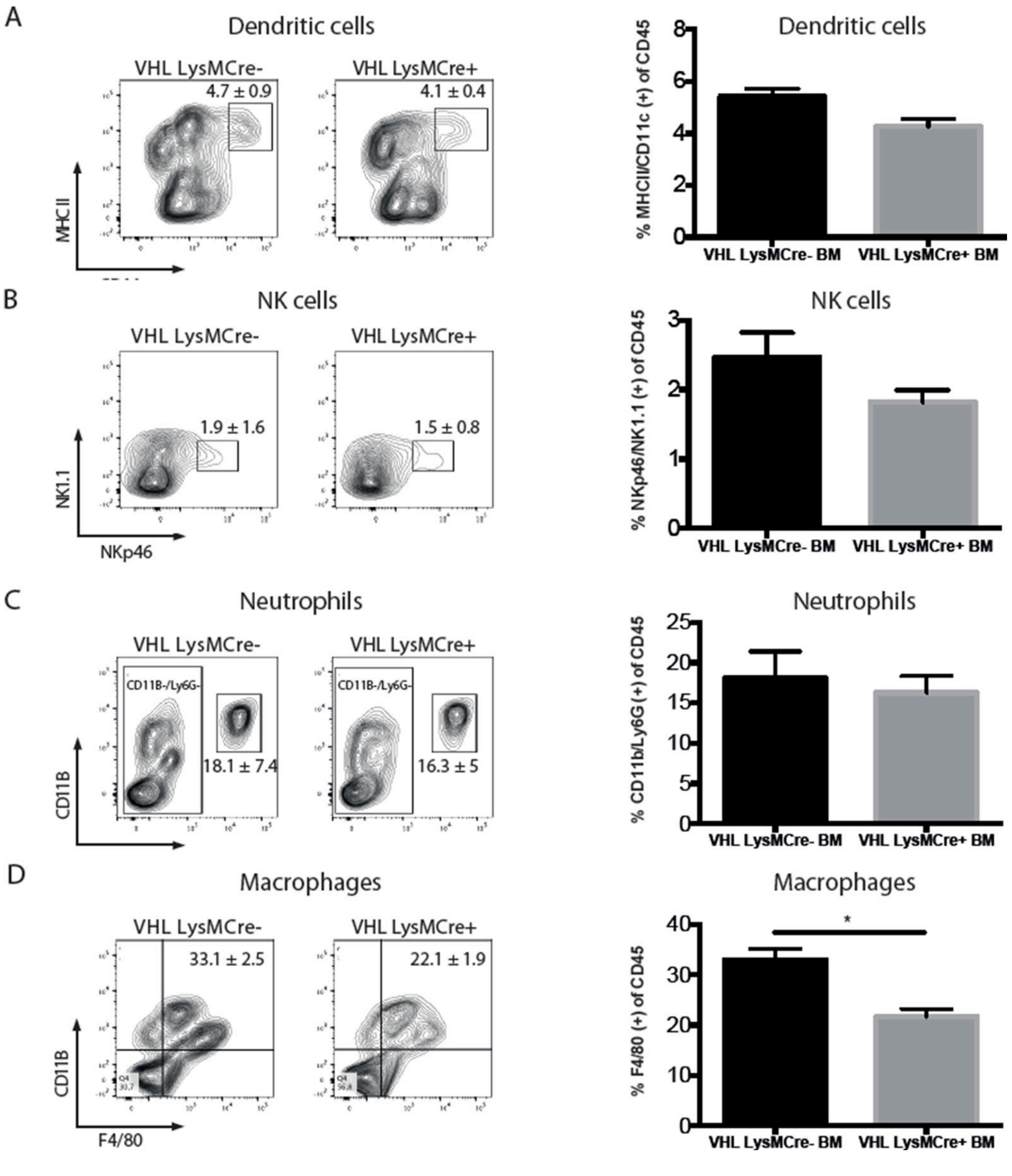

Supplementary Figure 2: Flow cytometric analysis and corresponding statistical analysis on whole livers 4 weeks after BM reconstitution showing percentages of: (A) viable CD45$^+$/MHCII$^+$/CD11C$^+$ dendritic cells. (**B**) viable CD45$^+$/NKp46$^+$/NK1.1+ NK cells. (**C**) viable CD45$^+$/CD11B$^+$/Ly6G$^+$ neutrophils. (**D**) viable CD45$^+$/Ly-6G-/CD3- /CD19-/NK1.1-/CD11B$^+$/F4/80+ macrophages. Error bars represent SEM (*n* = 5 for VHL LysMCre- and *n* = 6 for VHL LysMCre$^+$).

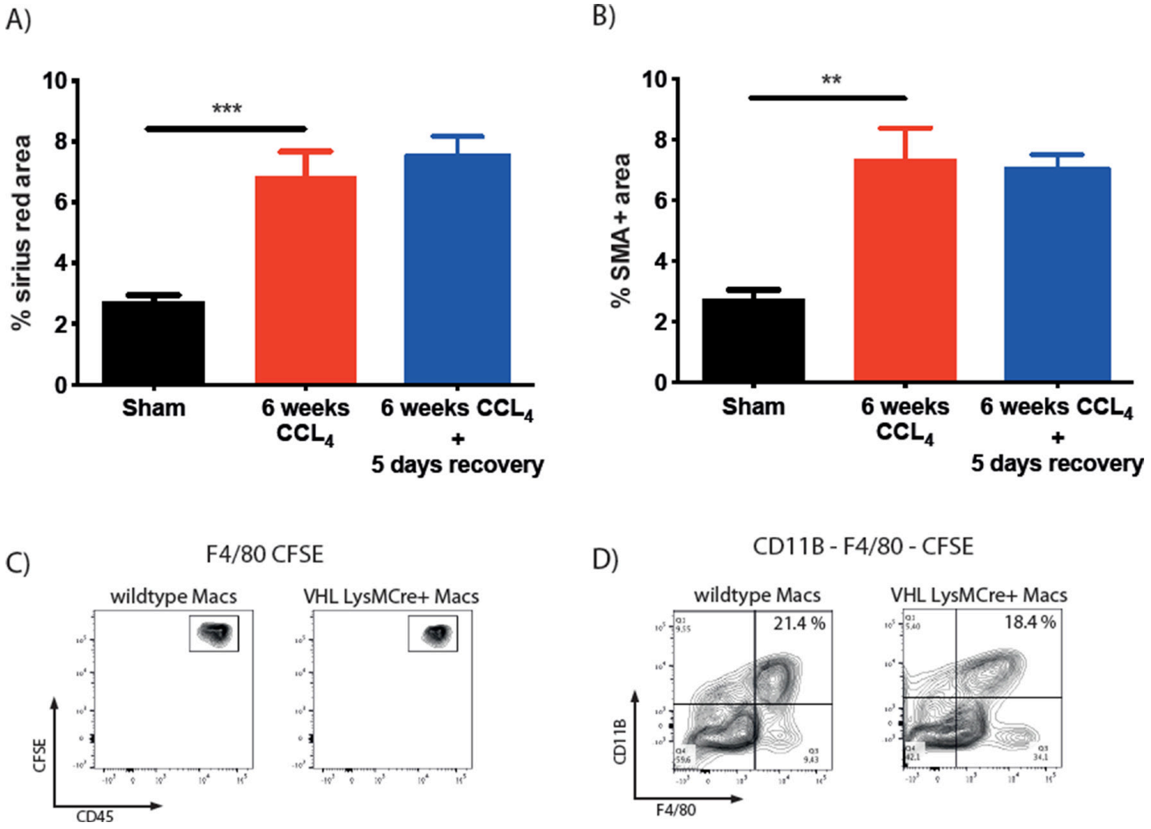

Supplementary Figure 3: (**A**) Quantification of Sirius Red-positive area on liver sections from mice after 6 weeks of sham-treatment, 6 weeks of CCL4 or CCL4 followed by 5 days of spontaneous recovery without macrophage therapy ($n = 5$). (**B**) Quantification of α—SMA-positive area on liver sections from mice after 6 weeks of sham-treatment, 6 weeks of CCL4 or CCL4 followed by 5 days of spontaneous recovery without macrophage therapy ($n = 5$). (**C**) Flow cytometric analysis on thioglycollate-elicited peritoneal cells ($CD45^+$) after CFSE-labelling ($n = 2$). (**D**) Flow cytometric analysis on dual positive CFSE/$CD45^+$ peritoneal cells for $CD11B^+$ $F4/80^+$ macrophages ($n = 2$). Error bars represent SEM.

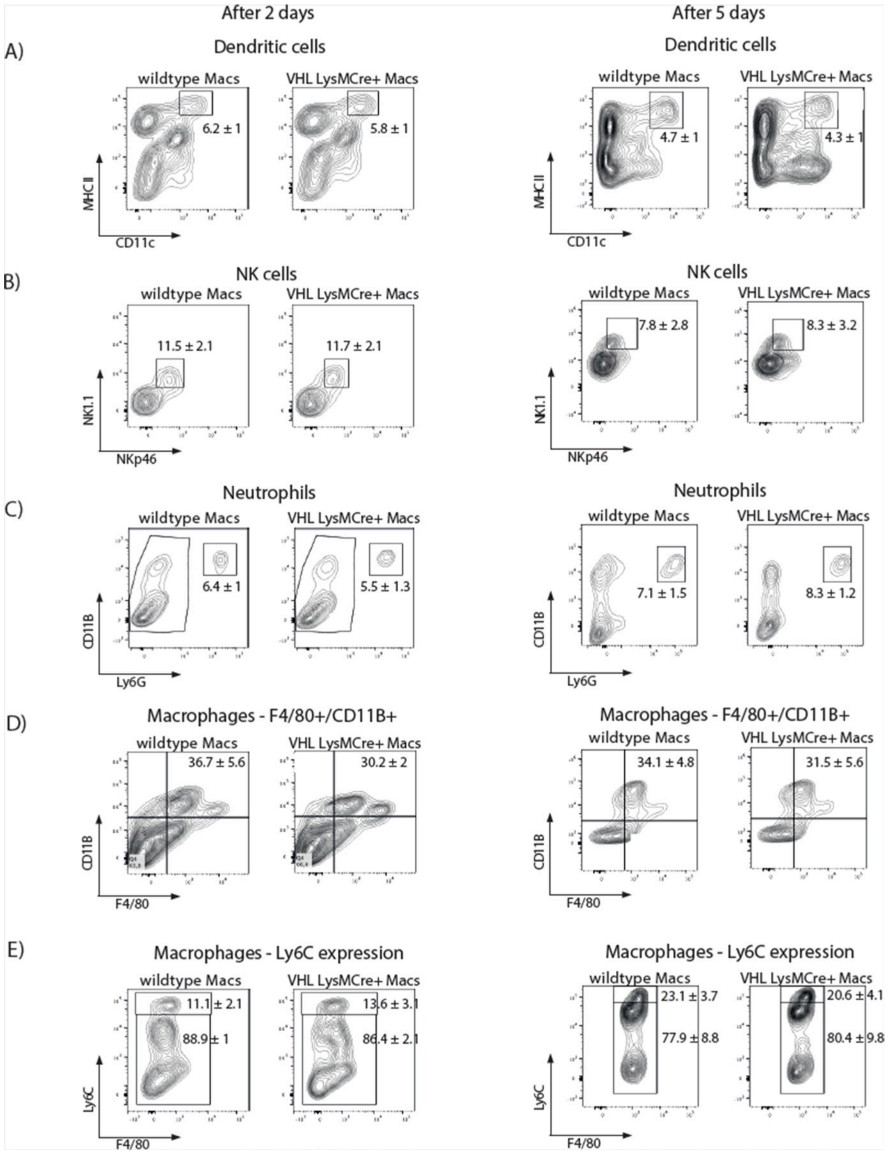

Supplementary Figure 4: Flow cytometric analysis on whole livers after macrophage infusion at the indicated time points for. (**A**) viable $CD45^+/MHCII^+/CD11C^+$ dendritic cells. (**B**) viable $CD45^+/NKp46^+/NK1.1^+$ NK cells. (**C**) viable $CD45^+/CD11B^+/$ Ly6G+ neutrophils. (**D**) viable $CD45^+/Ly\text{-}6G^-/CD3^-/CD19^-/NK1.1^-/CD11B^+/F4/80^+$ macrophages. (**E**) viable CD11Bhigh F4/80int Ly6Clo restorative macrophages. (*n* = 4 at day 2 and *n* = 5 at day 5 for wildtype Macs; *n* = 4 at day 2 and *n* = 6 at day 5 for VHL LysMCre+ Macs).

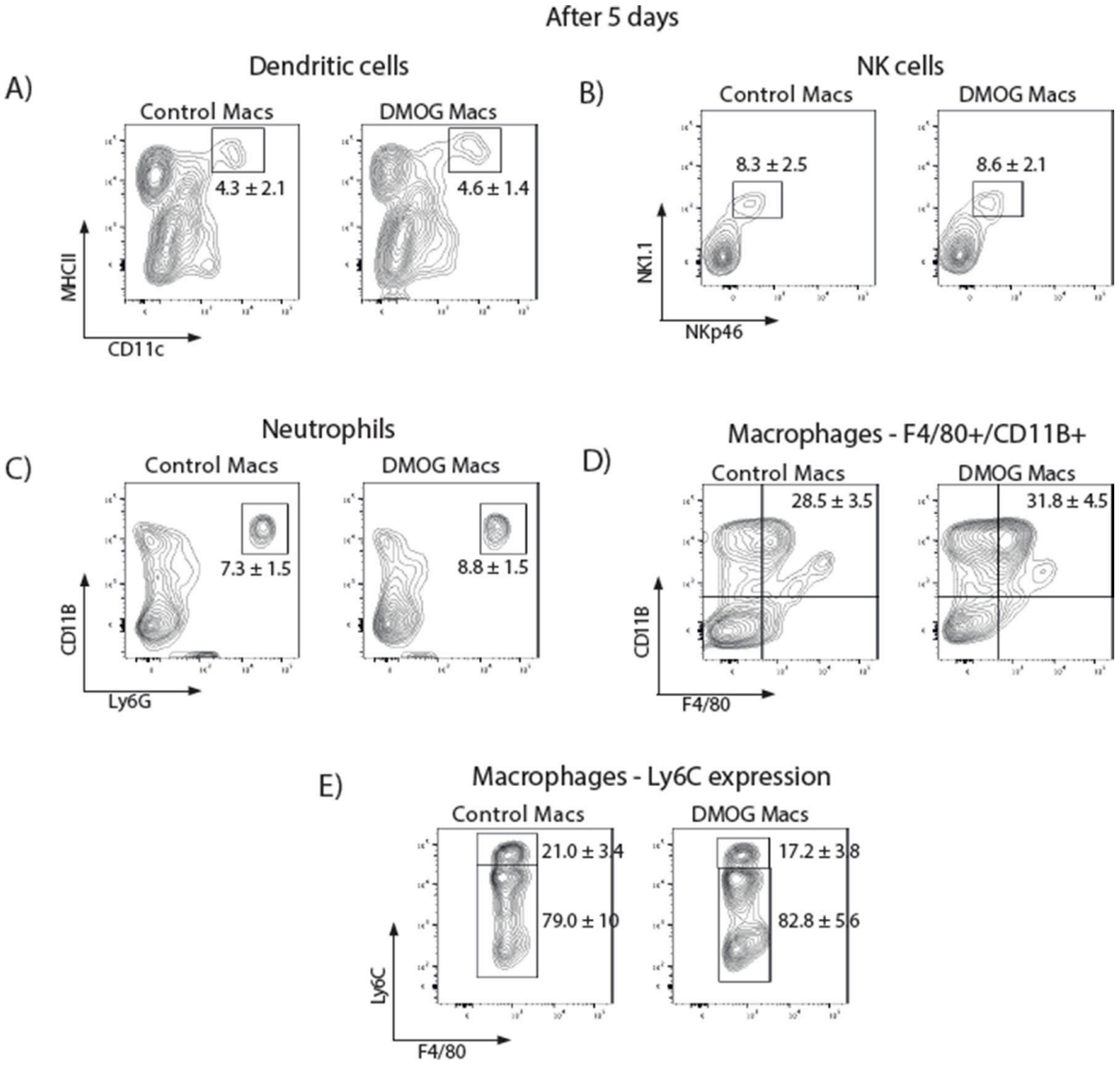

Supplementary Figure 5: Flow cytometric analysis on whole livers 5 days after infusion of DMOG-treated macrophages. (**A**) viable $CD45^+/MHCII^+/CD11C^+$ dendritic cells. (**B**) viable $CD45^+/NKp46^+/NK1.1^+$ NK cells. (**C**) viable $CD45^+/CD11B^+/Ly6G^+$neutrophils. (**D**) viable $CD45^+/Ly\text{-}6G^-/CD3^-/CD19^-/NK1.1^-/CD11B^+/F4/80^+$ macrophages. (**E**) viable CD11Bhigh F4/80int Ly6Clo restorative macrophages. (*n* = 7 for both treatment modalities)
